# Predatory journals and meetings in forensic sciences: what every expert needs to know about this “parasitic” publishing model

**DOI:** 10.1080/20961790.2021.1989548

**Published:** 2021-11-22

**Authors:** Ricardo Jorge Dinis-Oliveira

**Affiliations:** a TOXRUN – Toxicology Research Unit, University Institute of Health Sciences (IUCS), CESPU, CRL, Gandra, Portugal; b Department of Public Health and Forensic Sciences, and Medical Education, Faculty of Medicine, University of Porto, Porto, Portugal; c UCIBIO, REQUIMTE, Laboratory of Toxicology, Department of Biological Sciences, Faculty of Pharmacy, University of Porto, Porto, Portugal

**Keywords:** Forensic sciences; predatory open access journals, predatory meetings, Jeffrey Beall’s list, research integrity, peer review, scientific publishing

## Abstract

The emergence of the Internet has transformed all areas of society. This includes the universe of scientific publications, with several publishers now exclusively focusing on the electronic format and open access model while expanding to a megajournal scope. In this context, the pandemic of predatory open access journals (POAJs) and meetings are of grave concern to the academic and research community. This new shift within academia produces a variety of new victims; namely, the authors themselves. In turn, scientific knowledge is often discredited, with the public placing less trust in science. Now more than ever, performing research with integrity and selecting a journal in which to publish requires close attention and expertise. The “predatory movement” has developed increasingly sophisticated techniques for misleading people into believing what seem to be credible professional layouts and legitimate invitations. Initiatives such as the Jeffrey Beall’s list, the Cabell’s Scholarly Analytics and Think.Check.Submit offer some guidance to uncover the “parasitic” intervention of predatory journals and meetings, but specific education in this field is sorely needed. This work aims to review the main characteristics of predatory journals and meetings and to analyze this topic in the context of forensic and legal medicine research.

## Introduction

Since researchers need to publish to maintain their status or advance in their academic careers and increase their *h*-index scores, the pressure to publish has grown exponentially [1–3]. As in other sectors of society such as economics, the maxim of supply and demand applies here. The problem with scholarly journals is that the supply is so extensive that it becomes difficult to distinguish between journals that are worthy of attention and those that are better to avoid.

The current emphasis seems to be shifting too far in the direction of speed science rather than careful, robust research. This needs to be balanced, particularly since scientific results often drive regulatory agencies’ decisions. In the race to find treatments and vaccines for COVID-19, for example, it has become even more essential for society to be able to trust science as well as the drug companies seeking regulatory approval. Prestigious medical journals are also victims of misconduct during the COVID-19 pandemic, particularly due to ultrarapid scientific data publication in some cases [4–6]. Moreover, in trying to understand the scientific aspects of the disease, the public seems to be more interested in reading and reacting to scientific papers. This presents a challenge, since scientific data are typically presented in terms that require specialized knowledge and literacy. The COVID-19 pandemic has also brought atypical times for scientific publishing. Most journals are publishing COVID-19-related topics in an open access format, and the peer review process is saturated, in some cases failing in its capacity to scrutinize research [7].

In the context of scientific publishing, predatory open access journals (POAJs) are emerging as a critical topic of discussion. Numerous publications address problems associated with POAJs, proposing potential solutions and educational campaigns that could reduce the flow of manuscripts to such journals [8–12]. In fact, some authors have warned that we are facing the age of academic racketeering [13]. A 2015 study reported more than half a million articles published in POAJs [14]. As scientists, nearly every day we receive poorly written email invitations to publish in POAJs, usually unrelated to our subject matter expertise. In addition, when we receive an invitation, others with whom we frequently coauthor papers also receive the same message. In an astonishing study, a fictitious scientist named Dr. Anna O. Szust (*Oszust* means “fraud” in Polish) submitted a fake application for an editor position to 360 journals, a mix of legitimate titles and suspected POAJs. Forty predatory journals accepted her name as editor (i.e. “Dr. Fraud”), some within only a few hours of being contacted [15]. In another study, John Bohannon submitted obviously false articles related to cancer treatment, with fake authors and affiliations, to 304 scientific journals and obtained an acceptance rate of 51.6% [16]. Sadly, anyone can have almost anything published [10] with the advent of a dubious marketplace where science is for sale [8]. Despite measures taken, the number of articles in these pseudo-academic journals is still rapidly increasing [8, 11, 17]. Although they exist in all scientific areas, biomedicine seems to be most affected [18]. Particularly in the forensic sciences, I have received numerous invitations to publish articles in predatory journals competing for authors and revenue, most of them with names similar to those of respected publications, which deliberately causes confusion. Indeed, I have often stopped to read an invitation email from the *Journal of Forensic Science and Research* (jfsr.journal@bioijournals.com), given the fact that I am accustomed to paying attention to correspondence from contacts of the original and legitimate journal, *Forensic Sciences Research* (fsr@ssfjd.cn).

It is important for us to exercise caution as we seek to avoid confusing POAJ with genuine open access journals. Although POAJs advertise peer review, this scrutiny is typically absent, as can easily be detected by looking at the date of submission, peer review, and receipt of the acceptance letter. Since results and conclusions may be produced in such journals without the benefit of critique by actual experts, inaccurate data may drive court decisions on the part of those not familiar with such publications, leading to harmful repercussions for patients and society, as well as for political and economic realms.

This paper seeks to complement and update my initial reflection on research integrity [4] by reviewing the main characteristics of predatory journals and meetings, analyzing this issue in the context of forensic and legal medicine research.

## Methods

An exhaustive search was conducted in a range of databases to achieve cross-disciplinary coverage, including PubMed (US National Library of Medicine), Web of Science, Embase and Scopus and Google Scholar, and *via* Google to identify grey literature (those produced outside the traditional commercial/academic publishing channels). Approximately 95 documents were consulted, with 54 citations, which are listed in the References section of this review. A date limit was not applied, but most publications retrieved were released after 2012 when the term “predatory journal” entered the mainstream literature [19]. Multiple combinations of the following keywords were used: predatory journals/publisher, predatory meetings/conferences, forensic sciences, legal medicine, law, and justice. Retrieved journal articles, as well as books, general newspapers, and government documents, were also reviewed for possible additional publications related to this topic. Nevertheless, it is possible that some relevant documents were missed, especially due to the absence of universally accepted terms regarding POAJs. To reduce the effect of this limitation, articles written in different languages such as English, French, Spanish and Portuguese were searched.

## Predatory journals

No standard accepted definition of predatory publishing currently exists [20]. Moreover, reductionist terms to denote predatory journals such as “illegitimate journals” [21], “dark” journals [22], “open access journals with questionable marketing and peer review practices” [14] have appeared in the literature, increasing confusion regarding the nomenclature around this topic. It is therefore difficult for academic and research institutions to educate authors and to establish explicit policies to deter submissions to predatory journals, which typically include dubious open access journals where the costs associated with publishing, such as article processing charges (APC) are paid by the authors [23, 24]. This business model is becoming the most popular among publishers as opposed to the traditional subscription-based journals in which the reader pays for the content. The term “predatory journal” was first coined by Jeffrey Beall [19], an American librarian and scientist, to refer to the exploitative and fraudulent open access publishing model that applies APC without actually providing the peer review editorial services associated with legitimate journals [19, 25]. Jeffrey Beall also called these “counterfeit and dishonest journals”, noting their lack of transparency [19, 26]. He has also criticized the Directory of Open Access Journals (DOAJ) “for relying on data supplied by journal publishers to determine whether the journal in question should be included in the directory” [27]. As a sort of autobiography, Jeffrey Beall provided an overview of the history of POAJs, his scathing criticism, and the beginning of his emotional and professional involvement with this problem [9]. The author has created and maintained a publicly accessible list since January 2012 (the Beall’s list) in his blog entitled *Scholarly Open Access* aiming to summarize potential, possible, or probable predatory scholarly open access publishers using his own predefined criteria. The website was shut down in January 2017, reportedly in response to threats, lawsuits and pressure from his employer, the University of Colorado Denver, and out of fear for his job [9, 28]. Fortunately, the essence of his work continues in several websites, such as the https://beallslist.net/, which provides an updated version of Beall’s list [29], the Cabell’s Scholarly Analytics, which provides a Blacklist (questionable journals) and Whitelist (reputable journals) available for a fee in http://www2.cabells.com/ [30], and the Think.Check.Submit campaign to help researchers identify trusted journals and publishers for their research (https://thinkchecksubmit.org/) [31].

In a recent study of the topic, although the US was the most common of the reported publishers’ country, when addresses were checked using Google Maps and Google Street View, in almost 50% of cases, the locations were considered “unreliable” [32]. The commonly retrieved locations in these cases were residential houses in rural or peripheral areas, but also included markets, pharmacies, post offices, and restaurants. Declaring false addresses in the US, UK, Canada or Australia is a devious means used to try to improve credibility and attract researchers from high-income countries [14]. Indeed, these predators typically have “offices” in Pakistan, Malaysia, India, or Nigeria [19, 33]. Curiously, it was suggested that there are more “British Journals” based in Pakistan than in the UK [34]. Table 1 compiles a checklist of some of the major characteristics of POAJs.

**Table 1.** Primary reported characteristics of predatory open access journals [20]

No.	Main characteristics of predatory journals
1	Persuasive language *via* aggressive e-mail spamming tactics and promises of publication in a short time while offering fast-track services for extra fees. This should not be confused with calls for papers as legitimate marketing strategies from reputable journals and publishers
2	Letters are flattering, and their content typically mentions previous publications of the author in a particular field
3	Claim to have an impact factor (or use fake designations such as Cite Factor, General Impact Factor, Global Impact Factor, International Scientific Indexing and Scientific or Journal Impact Factor) or other indexes (e.g. Scopus, PubMd and DOAJ) that turn out to be false. It is important to verify the veracity of information in original websites such as the Journal Citation Reports of Clarivate®
4	APC are not explicit and usually claim generous discounts. Some demand APC before acceptance. Very low APC, such as <$150 USD are typically practiced
5	Lack transparency and publish virtually anything without quality peer review (i.e. guaranteed publishing), claiming “multidisciplinary scope” to attract authors from different fields
6	Manuscript corrections are usually not requested
7	The office address is not specific or accessible and may be a shop, park, or private apartment
8	Use “free e-mail” addresses (e.g. @gmail.com or @yahoo.com) rather than professional sources for correspondence; and article submissions occur *via* email and not online *via* specific platforms such as ScholarOne® and Editorial Manager®
9	Names and logos mimic reputable journals and typically include words such as “British”, “American”, “European”, etc. in the journal title
10	Some of these journals may exist for only a few weeks
11	Articles are published predominantly by authors around the world, but specially by authors from certain developing countries
12	Advertise that they are affiliated with COPE and WAME and follow ICMJE guidelines, but this is not the case; intentionally misrepresent their own practices
13	Editorial board is not clearly visible, or is incomplete and lacks legitimacy (e.g. appointed without knowledge, irrelevant skillset, and “serving” on boards of several journals of different scientific areas)
14	No digital preservation of articles, guidelines for determining authorship (e.g. ICMJE), or retraction policies for cases of misconduct
15	Journal is included in Jeffrey Beall’s or Standalone lists or has been flagged by Retraction Watch website
16	Articles are never cited in reputable journals
17	No mention of word limit for articles as the online maintenance costs are irrelevant

APC: article processing charges; DOAJ: Directory of Open Access Journals; WAME: The World Association of Medical Editors; ICMJE: International Committee of Medical Journal Editors; COPE: Committee on Publication Ethics.

Supplementing this list, Iowa State University attempted to classify predatory journals, proposing four different categories: phisher, imposter or hijacker, trojan horse, and unicorn. Definitions are provided in Table 2.

**Table 2.** Picturesque names given to predatory open access journals by Iowa State University (https://instr.iastate.libguides.com/predatory/id).

Picturesque names	Classification of predatory journals by Iowa State University
**Phisher**	Journals entice the author with promises of fast printing, but after acceptance, high APC, which are not previously mentioned either on the journal webpage or when the article was uploaded, are requested
**Imposter or Hijacker**	Journals try to look like well-known publishers, but there are additional words in their titles, such as “international”, “review”, etc. Hijackers usually have webpages that are deceptively similar in design and web address to credible journals
**Trojan Horse**	Journals that have a well-ordered website, often an impressive list of journals and articles, but such articles either do not exist, or worse, are stolen or plagiarized
**Unicorn**	Publishers who may run legitimate businesses but do not follow publisher recommendations, which could lead to ethical violations, an imperfect quality peer review process, or a lack of archiving policies, meaning that the article may disappear at any time

APC: article publishing charges.

Transformation of legitimate journals into predatory journals is also an important issue. Maintenance is relevant, because despite considerable efforts, sometimes even good journals cannot withstand the competition. For example, the esteemed Canadian medical journal, *Experimental & Clinical Cardiology*, after being sold by the Pulsus Publishing Group in 2013 to the offshore owners of Cardiology Academic Press, started to publish scientific “junk for hire”, still capitalizing on the journal’s original good name [35].

## Predatory meetings

Although not as pervasive as POAJs, the literature in this area also reveals the harmful impacts of predatory conferences [36, 37]. Jeffrey Beall first coined the term “predatory meetings/conferences” to characterize the activity of the OMICS Publishing Group and others in organizing scientific conferences claiming several characteristics, the majority of which are fake or fictional. This represents an expansion of the predatory publishing business model to exploit presenters and attendees only to maximize their profits [38] and certainly are not the authentic meetings the presenters and attendees anticipated [39, 40]. Like POAJs, predatory conferences promote the dissemination of questionable scientific information. Early career academics and researchers from developing countries are the most likely to be vulnerable to exploitation by predatory meetings, as is the case with POAJs, but the phenomenon also involves researchers from prestigious universities in high-income countries of Europe and North America [32, 41]. Although seasoned authors tend to instantly recognize the red flags of these amateur websites, this is not necessarily the case with more naïve authors. This issue may become even more flagrant as many scientists are keen to attend virtual meetings since the COVID-19 pandemic emerged, raising the possibility that more researchers will want to attend more meetings given the convenience of online portals [42]. Besides the Think.Check.Submit initiative described above, the Eaton’s list provides a comprehensive guideline to help determine the legitimacy of a conference through an algorithmic question format [43]. Although no forensic-specific lists of predatory meetings have been compiled yet, the generalist Caltech Library website, which provides a list of questionable conferences and conference organizers [44], describes several predatory forensic science conferences. Table 3 summarizes essential points that may help prevent falling for predatory conferences.

**Table 3.** Characteristics of predatory meetings/conferences [44].

No.	Predatory meetings/conferences
1	Use the names and photographs of prominent academics and scientists in organizing committees, often without their permission, to invite participants to their meetings which are falsely “signed” by members of the editorial boards
2	Promote their meetings in the same cities and with names very similar to other well-recognized and authentic meetings that have been occurring for years and linked to scientific societies
3	Not infrequently, the meetings are usually held in an airport hotel or in an attractive tourist venue
4	High fees for attendance and with no review standards for acceptance
5	Refuse to refund registration fees, even if the meetings are cancelled or postponed; instead, they may grant a credit for other “conferences”
6	Send invitations to authors for conferences outside the scope of their expertise
7	Language in emails is often too informal (e.g. “Dear Friend”, “Dear Esteemed Colleague”), in poorly written and unprofessional English terms
8	Organizers falsely claim that certain respected institutions, universities, and associations are their partners and sponsors
9	Accept submissions of poor quality and without obvious peer review within a week and even before the Call for Papers has closed
10	Conference organizers have links to predatory journals rather than respected scientific societies
11	Use subdomains hosted in generic website domains (e.g. xxx.conferences.com)
12	Technical and scientific programme is overly broad, seeking to attract various attendees
13	May alternate between countries, offering several meetings per year to increase profit
14	Websites and emails resemble travel and holiday brochures rather than scientific conferences

Reported organizers of predatory meetings include the World Academy of Science, Engineering and Technology (WASET) and the OMICS Publishing Group, but there are many other organizations offering the same deceptive kinds of meetings. On 25 August 2016 the US Federal Trade Commission (FTC) filed a lawsuit against the OMICS Publishing Group, iMedPub, ConferenceSeries, and Srinubabu Gedela (an Indian CEO of the companies) regarding its predatory journals and conferences [45]. In 2013, the US National Institutes of Health had stopped listing OMICS publications in PubMed Central and requested that this publisher stop making false claims of US government affiliations [46]. In March 2019, a US federal judge ruled that “OMICS made deceptive claims to academics and researchers about the nature of their conferences and publications” and ordered Srinubabu Gedela and his companies to pay $50.1 million in damages [45].

## Possible consequences for forensic sciences

The 21st century marks a turning point in evidence-based forensic sciences with the advent of several new techniques and research, in part due to the interest of researchers in this area, and possibly also triggered by the success of TV series such as “CSI”. Forensic experts tend to follow the published literature to handle our complex forensic diagnoses and cases. Nevertheless, accepting fake evidence could lead to erroneous judicial decisions, with detrimental effects on the focus on the person who is a victim of violence, which is, in broad sense, the major “object” of the forensic routine. Sometimes we must also discount and demystify aberrant theories that flourish in predatory forensic literature that has not been fully scrutinized. Such consequences can last for decades and have long-term global repercussions. These consequences can be even more harmful if the article was published in legitimate journals and then proved to be misconduct, as occurred with measles vaccination [24, 47, 48]. Those not aware of the drawbacks of bibliometrics may base their claims on “polluted theories” and “pseudoscientific evidence” simply because they are published in a “scientific journal”. Indeed, for a lawyer, judge or any other judicial professional, an article published by a POAJs can be almost undistinguishable from those produced by legitimate journals. From another perspective, a junior researcher who performs serious work, writes the scientific manuscript, and submits it unknowingly to a POAJs, will not receive the scientific recognition they deserve. Even worse, the junior researcher may see their name linked to dishonest publications, damaging their future career prospects and negatively affect the image and ranking of their countries and the chance of future publications in top journals. As a Department Director, I frequently review *curriculum vitae* for proposed integration in forensic academy and research institutions. I immediately scrutinize the source of the author’s publications, since I feel that if they are produced in predatory journals, something is wrong as far as the researcher’s integrity, credibility and scientific values [4].

In Portugal, we have a proverb that can be roughly translated as follows: “tell me what you got and where you got it and I will tell you how much it is worth”. In other words, without proper assessment of academic and research outputs of researchers, the truth of professional advancement may be distorted. One study demonstrated that the majority of faculty researchers had published in POAJs, and there was a positive correlation between predatory publications and receipt of internal research awards [49]. This paper did not assess whether poor quality data of POAJs have already been used to sustain court decisions. Post-publication peer review is raising awareness of the phenomenon and may contribute to introducing changes and positive reforms. Nevertheless, this review highlights that predatory publishing is prevalent in the broad field of forensics, as shown by the high number of retrieved publishers and journals in the archived but regularly updated versions of the original Jeffrey Beall’s list for publishers (last updated March 7, 2021) and of the Standalone list for journals not included in a publisher (last update February 5, 2021), both freely accessible online [29]. Although the exact number was not possible to estimate, POAJs seem to exceed the number of genuine forensic sciences journals. It should also be noted that many other journals not having “forensic”, “legal”, “law” or “justice” in their titles are publishing forensic results in specific areas such as toxicology, genetics, medicine, etc., suggesting that the actual number of POAJs in the field may be even higher.

Although typically POAJs become repositories for low quality scientific articles, it is possible that some legitimate articles occur within POAJs, since at the time of submission, the well-intentioned authors believed that they were dealing with a reputable journal. Forensic experts with a scientific background in bibliometrics are certainly more capable of producing higher quality forensic reports by selecting and interpreting published scientific results and avoiding counterfeit science.

## Conclusion

The open access model is a noble one. Still, it has rapidly paved the way for opportunistic predatory publishers who threaten the credibility of science, as claimed in a joint statement from three prominent medical writing societies [50]. This movement has also led to negative publicity for open access journals that use APC. Indeed, the payment of APC is a double-edged sword business model for journals, since such payment inevitably generates a conflict of interest, given that the journal may be incentivized financially to accept papers as a means of increasing revenue. Nevertheless, authors should be aware that many credible journals require an APC payment for legitimate reasons.

Publishing in and sitting on an editorial board of POAJs can damage a researcher’s reputation as well as that of their affiliated institution. As they embark on their careers, I always inform my students and junior faculty that after their first appearance in a scientific congress with an abstract or as an author of an article, they will be inundated with invitations to submit their work for publication in any number of “new” open access online journals. I encourage them to ignore devious invitations and implore them not to fall into the trap of this dark side of science [51, 52]. Besides using the lists described above, another excellent way to track POAJs or predatory conferences is by searching for their record on Google and cross-referencing their names with the word “predatory”. If the journal or conference is questionable, this search will likely reveal previous experiences shared by other authors, alerting naïve researchers to their perils.

Hijacked journals also represent a formidable threat to research integrity, abusing as POAJs, of the open access publishing model. The term “hijacked journals” is used to describe fake websites that mimic authentic and reputable journals and their websites, abusing both established names and identities such as the ISSN with the sole purpose of financial exploitation [53, 54]. They have a clearer criminal nature since the mechanism is typical of theft and/or robbery, while financial exploitation of the POAJs comes only from the APC.

Establishing a consensus definition of POAJs and predatory conferences could be a starting point for implementing policies and educational initiatives to reduce such submissions, particularly because of the vulnerability of young, inexperienced researchers who are eager to publish and suffering from anxiety to increase the number of publications in a short time, especially those coming from developing countries [41]. Junior researchers currently receive little education in research integrity, which is a serious problem. Such education should include lessons in how to use bibliometric tools, and support for navigating journal selection and submission processes [20]. Most important for an author considering publishing in a specific journal is scrutinizing the journal’s legitimacy and keeping eyes open for clues. Unfortunately, no list of POAJs and predatory meetings in forensic sciences research has yet been compiled. Since forensic researchers often publish in journals not specific to forensics, the general lists noted above offer a good starting point for revealing predatory practices seeking to attract authors in our field. In any case, authors, publishers, and universities should work together to improve the transparency and integrity of science.
